# Supplementary material for: Distinct mRNAs in Cancer Extracellular Vesicles Activate Angiogenesis and Alter Transcriptome of Vascular Endothelial Cells
Source: Cancers (Basel). 2021 Apr 22;13(9):2009. doi: 10.3390/cancers13092009 (PMC8122258; doi:10.3390/cancers13092009)
Supplement: Supplementary file 1 [file cancers-13-02009-s001.zip › anotated original western blot images.pdf]

Figure 1B

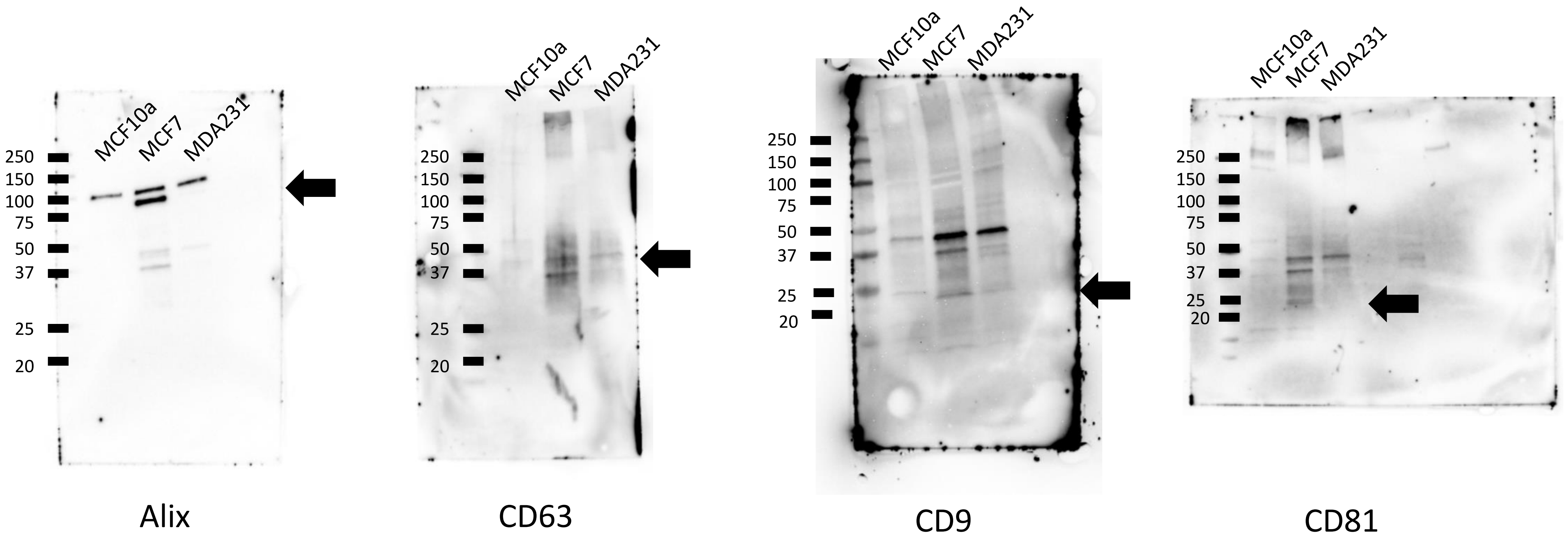
MCF10a
MCF7
MDA231
250
150
100
75
50
37
25
20
Alix
CD63
CD9
CD81

Figure 5D

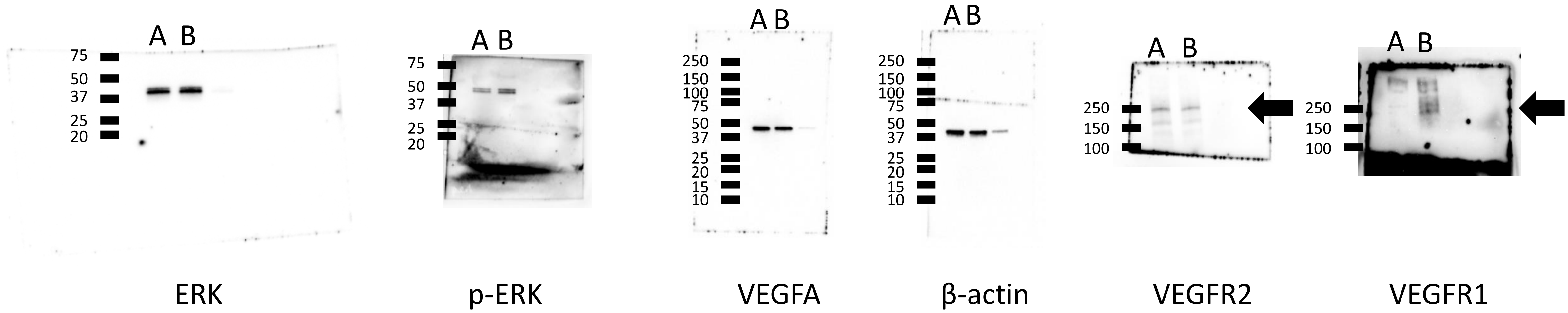

Sample in lane A: whole cell lysates of HUVECs not treated by MDA231-HEVs

Sample in lane B: whole cell lysates of HUVECs treated by MDA231-HEVs

Figure S3E

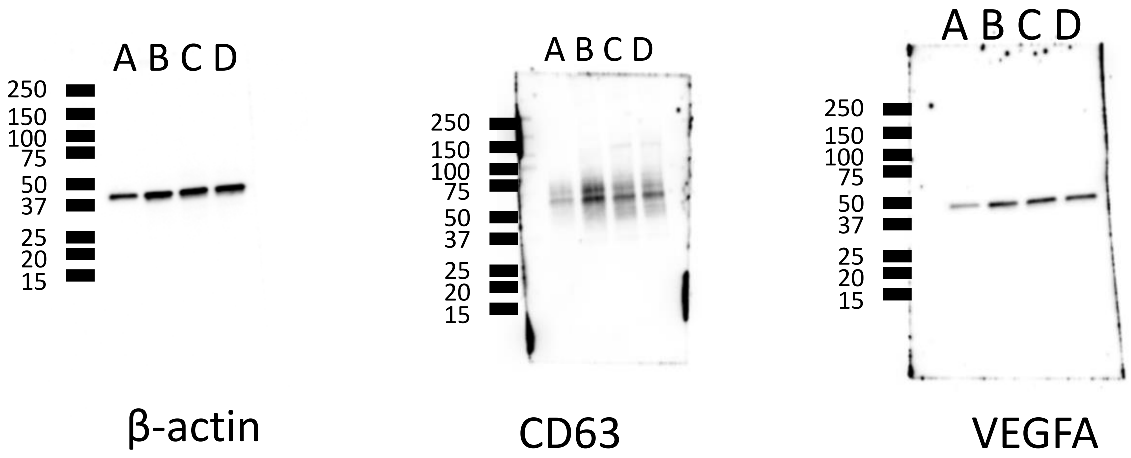

Sample in lane A: MDA231-NEVs

Sample in lane B: MDA231-HEVs

Sample in lane C: whole cell lysates of normoxic MDA231 cells

Sample in lane D: whole cell lysates of hypoxic MDA231 cells
