# Supplementary material for: Distinct mRNAs in Cancer Extracellular Vesicles Activate Angiogenesis and Alter Transcriptome of Vascular Endothelial Cells
Source: Cancers (Basel). 2021 Apr 22;13(9):2009. doi: 10.3390/cancers13092009 (PMC8122258; doi:10.3390/cancers13092009)
Supplement: Supplementary file 1 [file cancers-13-02009-s001.zip › Supplementary figures and table.pdf]

cancers

*Article*

# Supplementary Materials: Distinct mRNAs in Cancer Extracellular Vesicles Activate Angiogenesis and Alter Transcriptome of Vascular Endothelial Cells

**Pan Zhang, Su Bin Lim, Kuan Jiang, Ti Weng Chew, Boon Chuan Low and Chwee Teck Lim**

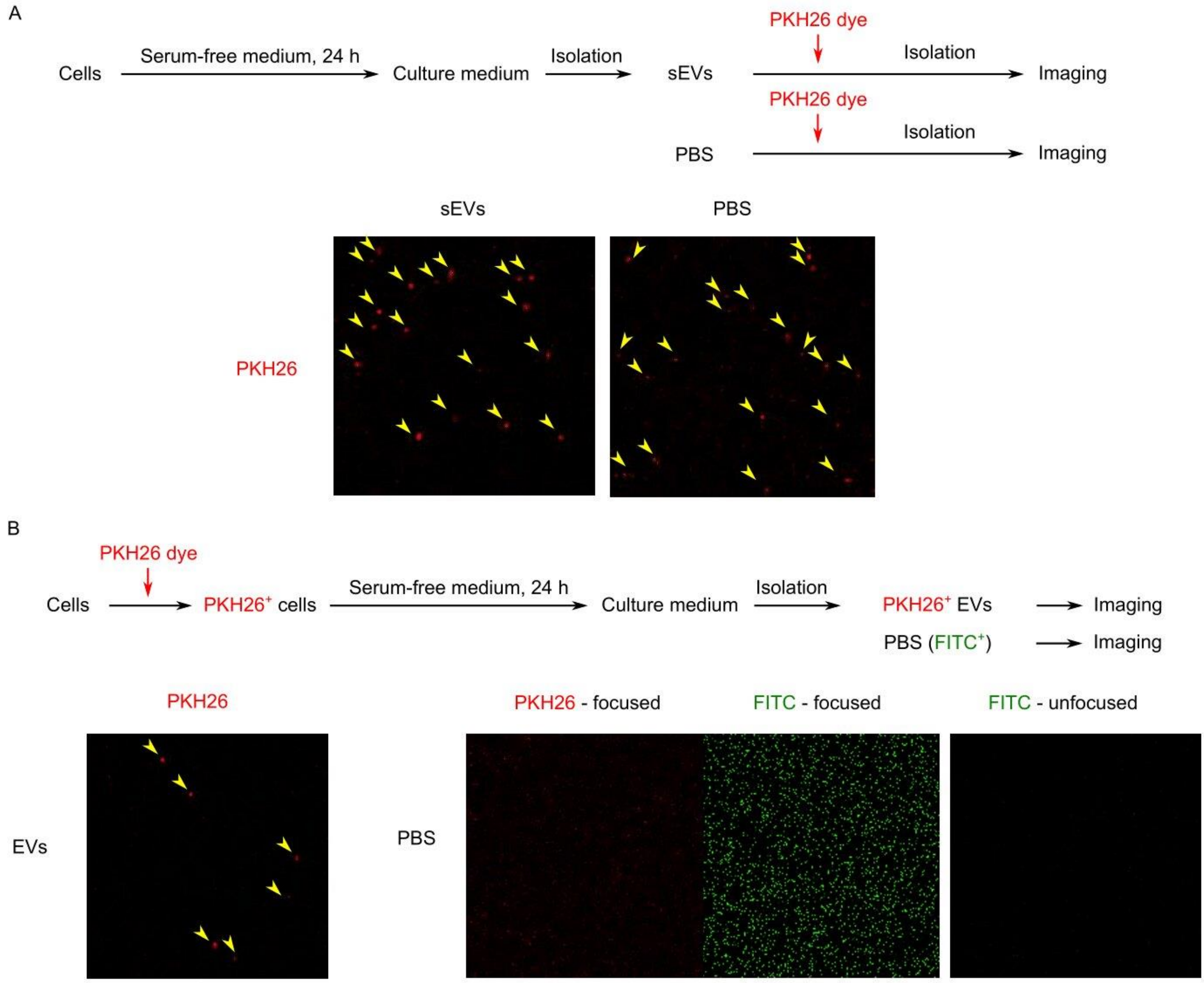


**Figure S1.** Comparison of two methods of EVs staining with PKH26. (**A**) Staining of EVs with direct mixing of EVs and PKH26 dye resulted in excessive dye that co-isolated with EVs and mimicked EVs in fluorescent imaging since abundant EV-like spots were identified in PBS control sample. (**B**) Cells that were stained with PKH26 dye generated PKH26$^+$ EVs. Excessive dye was thoroughly wash off after cell staining. PBS control showed no fluorescence.

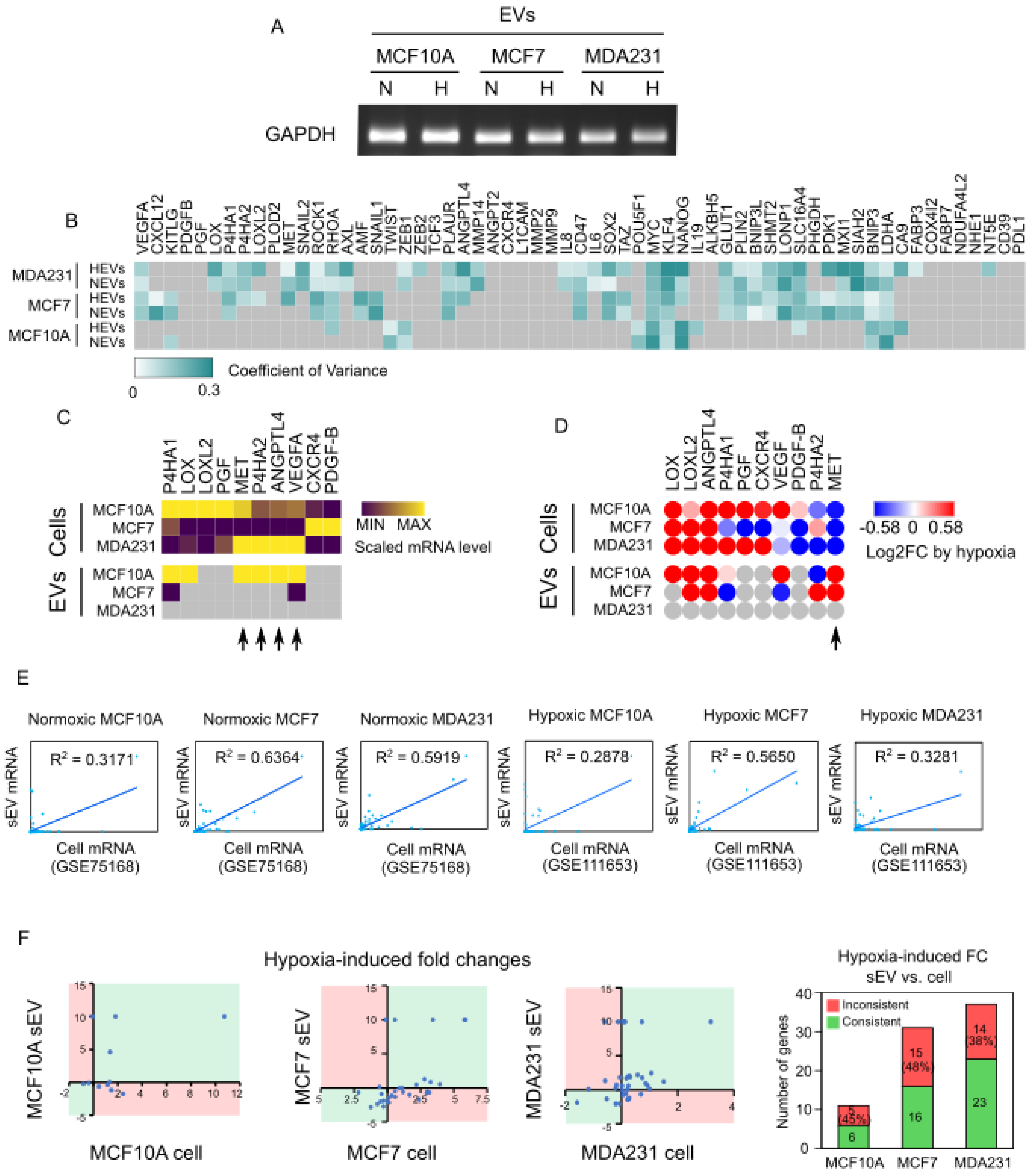


**Figure S2.** Comparison of mRNA profile between EVs and cells. (**A**) GAPDH mRNA was robustly present in all the quantified EVs. (**B**) The coefficient of variances of all gene expressions were less than 0.3, demonstrating that the exosomal mRNA levels were homeostatic, and the quantification of exosomal mRNA by RT-qPCR was reproducible. (**C**) Comparison of gene expressions in cells and EVs. The expression levels were normalized to GAPDH. (**D**) Comparison of hypoxia induced alterations of mRNA level in cells and EVs. (**E**) Expression correlation plot of hypoxia gene panel between EVs and cells. The R-squared values were calculated with linear regression. (**F**) The relationship of the hypoxia-induced gene expression fold change between EVs and cells.

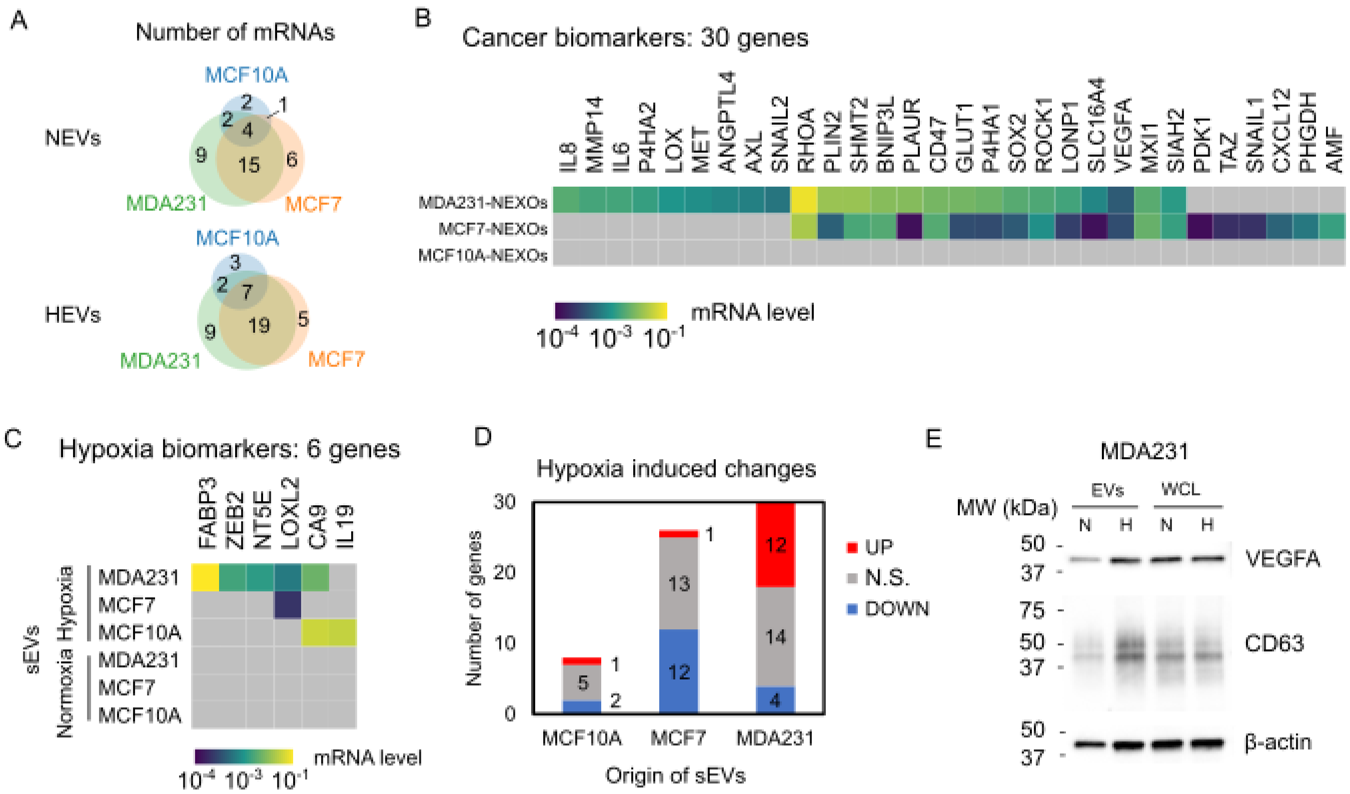


**Figure S3.** Analysis of exosomal mRNA profile of the hypoxia gene panel. (**A**) Venn diagram shows the presence of mRNAs of the hypoxia gene panel in normoxic EVs (NEVs) and hypoxic EVs (HEVs). (**B**) 30 genes from the hypoxia gene panel were potential cancer biomarker since their mRNAs were found present in cancer EVs while absent in MCF10A EVs. (**C**) 6 genes from the hypoxia gene panel can potentially reflect the hypoxic status of the tumor microenvironment as their mRNAs were only present in hypoxic EVs. (**D**) The number of mRNAs that were upregulated, downregulated or not significantly altered by hypoxic stress. Of the significantly regulated genes, most were downregulated in MCF7 and up-regulated in MDA231. (**E**) Western blot shows that the VEGFA protein level in cells (normalized to β-actin) and in EVs (normalized to CD63) were not changes by the hypoxic stimulus.

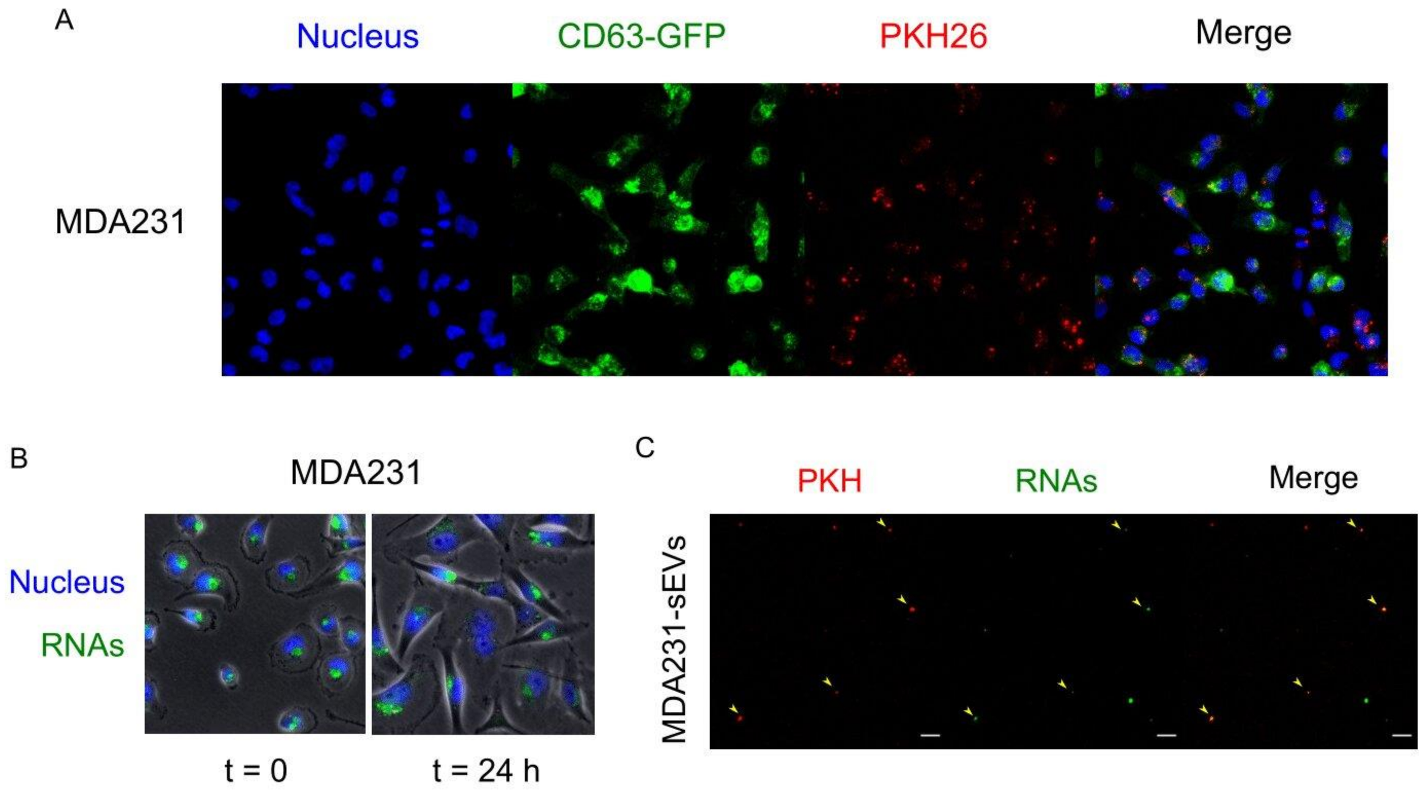


**Figure S4.** Fluorescent staining of CD63 and RNAs in MDA231 cell and EVs. (**A**) CD63-GFP and PKH26 efficiently label MDA231 cells. (**B**) The SYTO RNAselect specifically stained cell RNAs and lasted for more than 24 hours. (**C**) MDA231-derived PKH26$^{+}$ EVs carry RNAs as they can be stained by SYTO RNAselect.

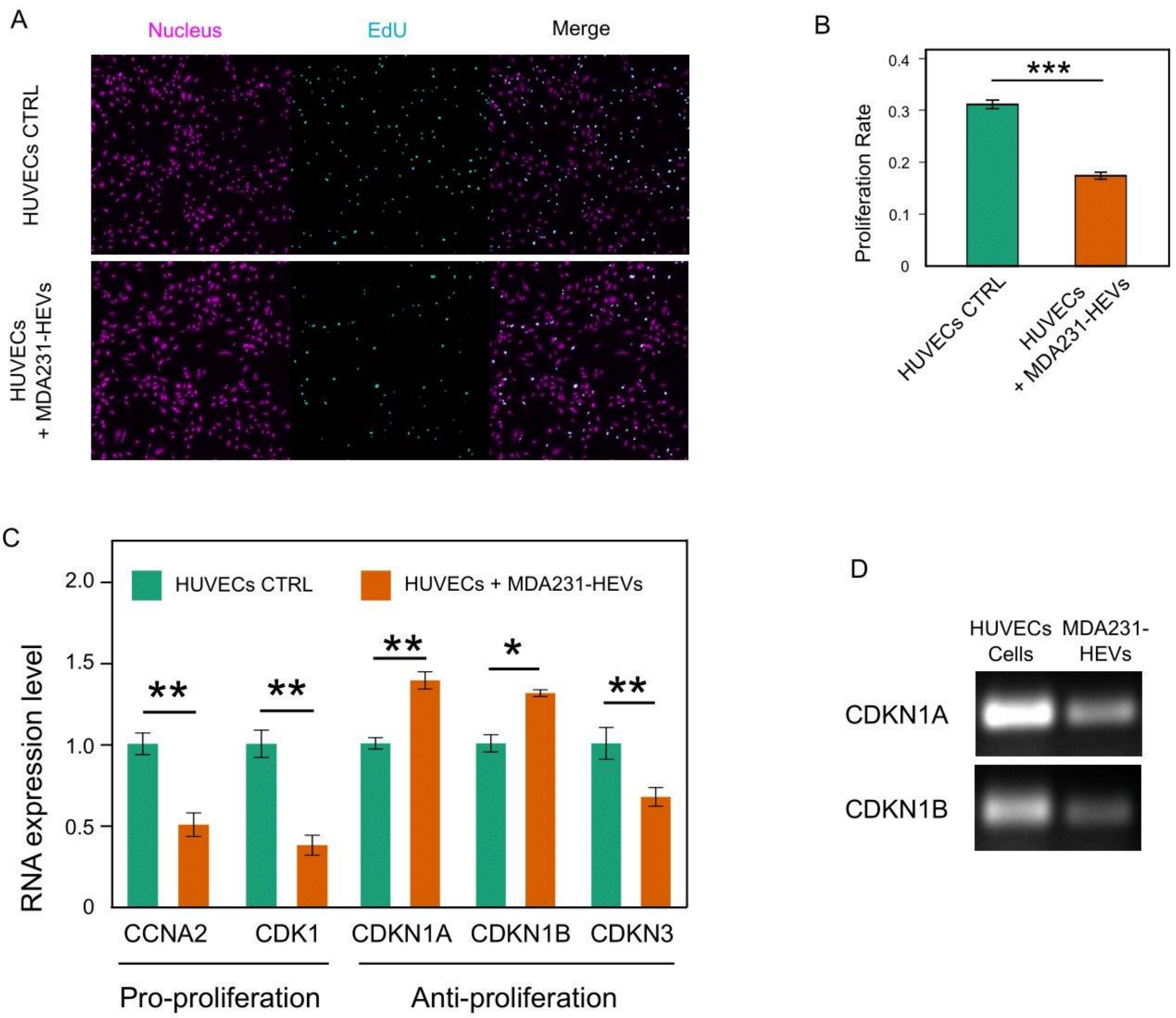


**Figure S5.** Cancer EVs suppress cell proliferation. (**A**) EdU proliferation assay of HUVECs in basal medium or treated with MDA231-HEVs for 24 h. (**B**) Proliferation rate of HUVECs were represented by the ratio of EdU+ cells to the Hoechst+ cells ($n = 25$). (**C**) The expressions of proliferation-related genes that were significantly altered by the treatment of MDA231-HEVs ($n = 3$). (**D**) The two upregulated CDK inhibitors, CDKN1A and CDKN1B, were carried by MDA231-HEVs. * $P < 0.05$, ** $P < 0.01$, *** $P < 0.001$ based on Mann-Whitney U test for (**B**), or Student's t test for (**C**). Error bars indicate the standard error of the mean (SEM) for (**B**) and (**C**).

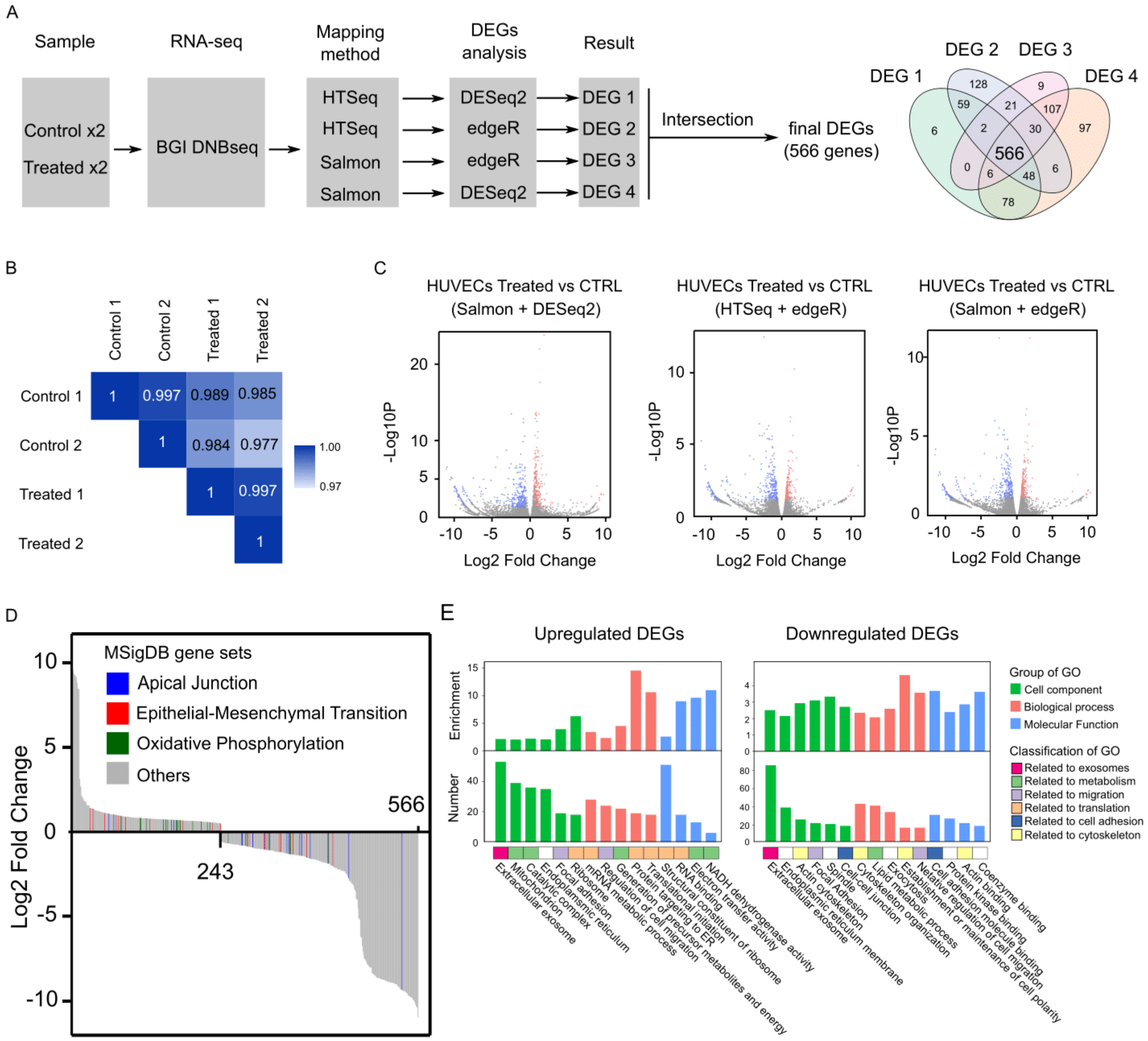


**Figure S6.** RNA-seq of HUVECs treated with cancer EVs. (**A**) Workflow of the RNA-seq data analysis. The final list of DEGs comprising 566 genes were acquired by intersecting four list of DEGs. (**B**) Correlation of four samples based on the expression profile shows the samples within each group were similar, while the correlation between control and EV-treated sample is relatively lower. (**C**) Volcano plot shows RNA-seq data of HUVECs with or without the treatment of MDA231-HEVs processed by pipelines of Salmon + DESeq2, HTSeq + edgeR, or Salmon + edgeR. (**D**) The plot of EV-induced fold changes of DEGs. Genes that belongs to Apical junction gene set, EMT gene set, or OP gene set were highlighted in blue, red, or green color, respectively. (**E**) Gene ontology analysis of DEGs. GO terms with >2 enrichment factor were ranked in gene number.

**Table S1.** Hypoxia-targeted gene panel.

| Gene Symbol in Paper | Official Gene Symbol | Official Full Name | Group of Function |
|---|---|---|---|
| **VEGFA** | VEGFA | vascular endothelial growth factor A | Angiogenesis |
| **CXCL12** | CXCL12 | C-X-C motif chemokine ligand 12 | Angiogenesis |
| **KITLG** | KITLG | KIT ligand | Angiogenesis |
| **PDGFB** | PDGFB | platelet derived growth factor subunit B | Angiogenesis |
| **PGF** | PGF | placental growth factor | Angiogenesis |

| | | | |
|---|---|---|---|
| **LOX** | LOX | lysyl oxidase | ECM stiffening |
| **P4HA1** | P4HA1 | prolyl 4-hydroxylase subunit alpha 1 | ECM stiffening |
| **P4HA2** | P4HA2 | prolyl 4-hydroxylase subunit alpha 2 | ECM stiffening |
| **LOXL2** | LOXL2 | lysyl oxidase like 2 | ECM stiffening |
| **PLOD2** | PLOD2 | procollagen-lysine,2-oxoglutarate 5-dioxygenase 2 | ECM stiffening |
| **MET** | MET | MET proto-oncogene, receptor tyrosine kinase | Cell motility |
| **SNAIL2** | SNAI2 | snail family transcriptional repressor 2 | Cell motility |
| **ROCK1** | ROCK1 | Rho associated coiled-coil containing protein kinase 1 | Cell motility |
| **RHOA** | RHOA | ras homolog family member A | Cell motility |
| **AXL** | AXL | AXL receptor tyrosine kinase | Cell motility |
| **AMF** | GPI | glucose-6-phosphate isomerase | Cell motility |
| **SNAIL1** | SNAI1 | snail family transcriptional repressor 1 | Cell motility |
| **TWIST** | TWIST1 | twist family bHLH transcription factor 1 | Cell motility |
| **ZEB1** | ZEB1 | zinc finger E-box binding homeobox 1 | Cell motility |
| **ZEB2** | ZEB2 | zinc finger E-box binding homeobox 2 | Cell motility |
| **TCF3** | TCF3 | transcription factor 3 | Cell motility |
| **PLAUR** | PLAUR | plasminogen activator, urokinase receptor | Metastasis |
| **ANGPTL4** | ANGPTL4 | angiopoietin like 4 | Metastasis |
| **MMP14** | MMP14 | matrix metallopeptidase 14 | Metastasis |
| **ANGPT2** | ANGPT2 | angiopoietin 2 | Metastasis |
| **CXCR4** | CXCR4 | C-X-C motif chemokine receptor 4 | Metastasis |
| **L1CAM** | L1CAM | L1 cell adhesion molecule | Metastasis |
| **MMP2** | MMP2 | matrix metallopeptidase 2 | Metastasis |
| **MMP9** | MMP9 | matrix metallopeptidase 9 | Metastasis |
| **IL8** | CXCL8 | C-X-C motif chemokine ligand 8 | Cancer stem cell maintainance |
| **CD47** | CD47 | CD47 molecule | Cancer stem cell maintainance |
| **IL6** | IL6 | interleukin 6 | Cancer stem cell maintainance |
| **SOX2** | SOX2 | SRY-box transcription factor 2 | Cancer stem cell maintainance |
| **TAZ** | TAZ | tafazzin | Cancer stem cell maintainance |
| **POU5F1** | POU5F1 | POU class 5 homeobox 1 | Cancer stem cell maintainance |
| **MYC** | MYC | MYC proto-oncogene, bHLH transcription factor | Cancer stem cell maintainance |
| **KLF4** | KLF4 | Kruppel like factor 4 | Cancer stem cell maintainance |
| **NANOG** | NANOG | Nanog homeobox | Cancer stem cell maintainance |
| **IL19** | IL19 | interleukin 19 | Cancer stem cell maintainance |
| **ALKBH5** | ALKBH5 | alkB homolog 5, RNA demethylase | Cancer stem cell maintainance |
| **GLUT1** | SLC2A1 | solute carrier family 2 member 1 | Metabolism |
| **PLIN2** | PLIN2 | perilipin 2 | Metabolism |
| **BNIP3L** | BNIP3L | BCL2 interacting protein 3 like | Metabolism |
| **SHMT2** | SHMT2 | serine hydroxymethyltransferase 2 | Metabolism |
| **LONP1** | LONP1 | lon peptidase 1, mitochondrial | Metabolism |
| **SLC16A4** | SLC16A4 | solute carrier family 16 member 4 | Metabolism |
| **PHGDH** | PHGDH | phosphoglycerate dehydrogenase | Metabolism |
| **PDK1** | PDK1 | pyruvate dehydrogenase kinase 1 | Metabolism |
| **MXI1** | MXI1 | MAX interactor 1, dimerization protein | Metabolism |
| **SIAH2** | SIAH2 | siah E3 ubiquitin protein ligase 2 | Metabolism |
| **BNIP3** | BNIP3 | BCL2 interacting protein 3 | Metabolism |
| **LDHA** | LDHA | lactate dehydrogenase A | Metabolism |
| **CA9** | CA9 | carbonic anhydrase 9 | Metabolism |
| **FABP3** | FABP3 | fatty acid binding protein 3 | Metabolism |
| **COX4I2** | COX4I2 | cytochrome c oxidase subunit 4I2 | Metabolism |
| **FABP7** | FABP7 | fatty acid binding protein 7 | Metabolism |
| **NDUFA4L2** | NDUFA4L2 | NDUFA4 mitochondrial complex associated like 2 | Metabolism |
| **NHE1** | SLC9A1 | solute carrier family 9 member A1 | Metabolism |
| **NT5E** | NT5E | 5'-nucleotidase ecto | Immune evasion |
| **CD39** | ENTPD1 | ectonucleoside triphosphate diphosphohydrolase 1 | Immune evasion |
| **PDL1** | CD274 | CD274 molecule | Immune evasion |

**Table S2.** Primer sequences for RT-qPCR.

| Gene Symbol | Forward | Reverse | Product Length (bp) |
|---|---|---|---|
| **Hypoxia-targeted gene panel** | | | |
| **ALKBH5** | 5'-CGGCGAAGGCTACACTTACG-3' | 5'-CCACCAGCTTTTGGATCACCA-3' | 128 |
| **AMF** | 5'-CCGCGTCTGGTATGTCTCC-3' | 5'-CCTGGGTAGTAAAGGTCTTGGA-3' | 113 |
| **ANGPT2** | 5'-ACCCCACTGTTGCTAAAGAAGA-3' | 5'-CCATCCTCACGTCGCTGAATA-3' | 184 |
| **ANGPTL4** | 5'-GGCTCAGTGGACTTCAACCG-3' | 5'-CCGTGATGCTATGCACCTTCT-3' | 103 |
| **AXL** | 5'-CCGTGGACCTACTCTGGCT-3' | 5'-CCTTGGCGTTATGGGCTTC-3' | 132 |
| **BNIP3** | 5'-CAGGGCTCCTGGGTAGAACT-3' | 5'-CTACTCCGTCCAGACTCATGC-3' | 131 |
| **BNIP3L** | 5'-TTGGATGCACAACATGAATCAGG-3' | 5'-TCTTCTGACTGAGAGCTATGGTC-3' | 140 |
| **CA9** | 5'-GGATCTACCTACTGTTGAGGCT-3' | 5'-CATAGCGCCAATGACTCTGGT-3' | 98 |
| **CD39** | 5'-AGGTGCCTATGGCTGGATTAC-3' | 5'-CCAAAGCTCCAAAGGTTTCCT-3' | 116 |
| **CD47** | 5'-TCCGGTGGTATGGATGAGAAA-3' | 5'-ACCAAGGCCAGTAGCATTCTT-3' | 129 |
| **COX4I2** | 5'-CCGTCGCTCCAATGAGTGG-3' | 5'-CGTCCGTCAAGGTGATCGG-3' | 125 |
| **CXCL12** | 5'-ATTCTCAACACTCCAAACTGTGC-3' | 5'-ACTTTAGCTTCGGGTCAATGC-3' | 88 |
| **CXCR4** | 5'-ACGCCACCAACAGTCAGAG-3' | 5'-AGTCGGGAATAGTCAGCAGGA-3' | 96 |
| **FABP3** | 5'-TGGAGTTCGATGAGACAACAGC-3' | 5'-CTCTTGCCCGTCCCATTTCTG-3' | 101 |
| **FABP7** | 5'-GCACATTCAAGAACACGGAGA-3' | 5'-CACATCACCAAAAGTAAGGGTCA-3' | 203 |
| **GLUT1** | 5'-ATTGGCTCCGGTATCGTCAAC-3' | 5'-GCTCAGATAGGACATCCAGGGTA-3' | 174 |
| **IL19** | 5'-TCCTGGCGTTCTACGTGGA-3' | 5'-TGACATTGCCGCAGAGTTTTC-3' | 124 |
| **IL6** | 5'-ACTCACCTCTTCAGAACGAATTG-3' | 5'-CCATCTTTGGAAGGTTCAGGTTG-3' | 149 |
| **IL8** | 5'-ACTGAGAGTGATTGAGAGTGGAC-3' | 5'-AACCCTCTGCACCCAGTTTTC-3' | 112 |
| **KITLG** | 5'-AATCCTCTCGTCAAAACTGAAGG-3' | 5'-CCATCTCGCTTATCCAACAATGA-3' | 163 |
| **KLF4** | 5'-CGGACATCAACGACGTGAG-3' | 5'-GACGCCTTCAGCACGAACT-3' | 139 |
| **L1CAM** | 5'-TGTCATCACGGAACAGTCTCC-3' | 5'-CTGGCAAAGCAGCGGTAGAT-3' | 246 |
| **LDHA** | 5'-TTGACCTACGTGGCTTGGAAG-3' | 5'-GGTAACGGAATCGGGCTGAAT-3' | 91 |
| **LONP1** | 5'-GACGATCCCCGATGTGTTTCC-3' | 5'-GGGCGAGACGAACTTTCCTT-3' | 137 |
| **LOX** | 5'-CGGCGGAGGAAAACTGTCT-3' | 5'-TCGGCTGGGTAAGAAATCTGA-3' | 128 |
| **LOXL2** | 5'-GGGTGGAGGTGTACTATGATGG-3' | 5'-CTTGCCCGTAGGAGGAGCTG-3' | 137 |
| **MET** | 5'-AGCAATGGGGAGTGTAAAGAGG-3' | 5'-CCCAGTCTTGTACTCAGCAAC-3' | 198 |
| **MMP14** | 5'-CATCTGTGACGGGAACTTTGA-3' | 5'-GGCAGTGTTGATGGACGCA-3' | 163 |
| **MMP2** | 5'-GATACCCCTTTGACGGTAAGGA-3' | 5'-CCTTCTCCCAAGGTCCATAGC-3' | 112 |
| **MMP9** | 5'-GGGACGCAGACATCGTCATC-3' | 5'-TCGTCATCGTCGAAATGGGC-3' | 139 |
| **MXI1** | 5'-GCGCCTTTGTTTAGAACGCTT-3' | 5'-AATGCTGTCCATTCGTATTCGT-3' | 235 |
| **MYC** | 5'-GGCTCCTGGCAAAAGGTCA-3' | 5'-CTGCGTAGTTGTGCTGATGT-3' | 119 |
| **NANOG** | 5'-TTTGTGGGCCTGAAGAAAACT-3' | 5'-AGGGCTGTCCTGAATAAGCAG-3' | 116 |
| **NDUFA4L2** | 5'-ATGATCGGCTTAATCTGCCTG-3' | 5'-TCCGGGTTGTTCTTTCTGTCC-3' | 104 |
| **NHE1** | 5'-ACCACGAGAACGCTCGATTG-3' | 5'-ACGTGTGTGTAGTCGATGCC-3' | 144 |
| **NT5E** | 5'-CCAGTACCAGGGCACTATCTG-3' | 5'-TGGCTCGATCAGTCCTTCCA-3' | 136 |
| **P4HA1** | 5'-CCAAAGCTCTGTTACGTCTCC-3' | 5'-AGTCCTCAGCCGTTAGAAAAGA-3' | 108 |
| **P4HA2** | 5'-CAAACTGGTGAAGCGGCTAAA-3' | 5'-GCACAGAGAGGTTGGCGATA-3' | 95 |
| **PDGFB** | 5'-CTCGATCCGCTCCTTTGATGA-3' | 5'-CGTTGGTGCGGTCTATGAG-3' | 239 |
| **PDK1** | 5'-GAGAGCCACTATGGAACACCA-3' | 5'-GGAGGTCTCAACACGAGGT-3' | 187 |
| **PDL1** | 5'-TGGCATTTGCTGAACGCATTT-3' | 5'-TGCAGCCAGGTCTAATTGTTTT-3' | 120 |
| **PGF** | 5'-GAACGGCTCGTCAGAGGTG-3' | 5'-ACAGTGCAGATTCTCATCGCC-3' | 187 |
| **PHGDH** | 5'-CTGCGGAAAGTGCTCATCAGT-3' | 5'-TGGCAGAGCGAACAATAAGGC-3' | 154 |
| **PLAUR** | 5'-TGTAAGACCAACGGGGATTGC-3' | 5'-AGCCAGTCCGATAGCTCAGG-3' | 166 |
| **PLIN2** | 5'-TTGCAGTTGCCAATACCTATGC-3' | 5'-CCAGTCACAGTAGTCGTCACA-3' | 148 |
| **PLOD2** | 5'-TTATTGAGCAACCAACCCCTTT-3' | 5'-GGCTTCCGCTTGACTTAGATTT-3' | 206 |
| **POU5F1** | 5'-GGGAGATTGATAACTGGTGTGTT-3' | 5'-GTGTATATCCCAGGGTGATCCTC-3' | 144 |
| **RHOA** | 5'-GGAAAGCAGGTAGAGTTGGCT-3' | 5'-GGCTGTCGATGGAAAAACACAT-3' | 118 |
| **ROCK1** | 5'-GGTGGTCGGTTGGGGTATTTT-3' | 5'-CGCCCTAACCTCACTTCCC-3' | 196 |
| **SHMT2** | 5'-CCCTTCTGCAACCTCACGAC-3' | 5'-TGAGCTTATAGGGCATAGACTCG-3' | 135 |
| **SIAH2** | 5'-CGCCAGAAGTTGAGCTGCT-3' | 5'-TGGTGGCATACTTACAGGGAA-3' | 115 |
| **SLC16A4** | 5'-AGGTATCCTTGAGACGGTCAG-3' | 5'-CAAGCAGGTTAGTGATGCCG-3' | 119 |
| **SNAIL1** | 5'-TCGGAAGCCTAACTACAGCGA-3' | 5'-AGATGAGCATTGGCAGCGAG-3' | 140 |

| | | | |
|---|---|---|---|
| **SNAIL2** | 5'-TGTGACAAGGAATATGTGAGCC-3' | 5'-TGAGCCCTCAGATTTGACCTG-3' | 203 |
| **SOX2** | 5'-GCCGAGTGGAAACTTTTGTCG-3' | 5'-GGCAGCGTGTACTTATCCTTCT-3' | 155 |
| **TAZ** | 5'-CACCGTGTCCAATCACCAGTC-3' | 5'-TCCAACGCATCAACTTCAGGT-3' | 95 |
| **TCF3** | 5'-ACGAGCGTATGGGCTACCA-3' | 5'-GTTATTGCTTGAGTGATCCGGG-3' | 233 |
| **TWIST** | 5'-GTCCGCAGTCTTACGAGGAG-3' | 5'-GCTTGAGGGTCTGAATCTTGCT-3' | 156 |
| **VEGFA** | 5'-AGGGCAGAATCATCACGAAGT-3' | 5'-AGGGTCTCGATTGGATGGCA-3' | 75 |
| **ZEB1** | 5'-CAGCTTGATACCTGTGAATGGG-3' | 5'-TATCTGTGGTCGTGTGGGACT-3' | 106 |
| **ZEB2** | 5'-CAAGAGGCGCAAACAAGCC-3' | 5'-GGTTGGCAATACCGTCATCC-3' | 128 |
| **Cell cycle-related genes** | | | |
| **CCNA1** | 5'-ACATGGATGAACTAGAGCAGGG-3' | 5'-GAGTGTGCCGGTGTCTACTT-3' | 95 |
| **CCNA2** | 5'-CGCTGGCGGTACTGAAGTC-3' | 5'-GAGGAACGGTGACATGCTCAT-3' | 120 |
| **CCNB1** | 5'-CCTGCCTGCAACAGTACCC-3' | 5'-CCAACACGATCTCTGGTCGC-3' | 115 |
| **CCND1** | 5'-GCTGCGAAGTGGAAACCATC-3' | 5'-CCTCCTTCTGCACACATTTGAA-3' | 135 |
| **CCND2** | 5'-ACCTTCCGCAGTGCTCCTA-3' | 5'-CCCAGCCAAGAAACGGTCC-3' | 161 |
| **CCND3** | 5'-TACCCGCCATCCATGATCG-3' | 5'-AGGCAGTCCACTTCAGTGC-3' | 128 |
| **CCNE1** | 5'-ACTCAACGTGCAAGCCTCG-3' | 5'-GCTCAAGAAAGTGCTGATCCC-3' | 141 |
| **CCNE2** | 5'-TCAAGACGAAGTAGCCGTTTAC-3' | 5'-TGACATCCTGGGTAGTTTTCCTC-3' | 115 |
| **CDK1** | 5'-AAACTACAGGTCAAGTGGTAGCC-3' | 5'-TCCTGCATAAGCACATCCTGA-3' | 148 |
| **CDK2** | 5'-CCAGGAGTTACTTCTATGCCTGA-3' | 5'-TTCATCCAGGGGAGGTACAAC-3' | 90 |
| **CDK4** | 5'-ATGGCTACCTCTCGATATGAGC-3' | 5'-CATTGGGGACTCTCACACTCT-3' | 124 |
| **CDK6** | 5'-CCAGATGGCTCTAACCTCAGT-3' | 5'-AACTTCCACGAAAAAGAGGCTT-3' | 152 |
| **CDK7** | 5'-ATGGCTCTGGACGTGAAGTCT-3' | 5'-GCGACAATTTGGTTGGTGTTC-3' | 116 |
| **CDKN1A** | 5'-TGTCCGTCAGAACCCATGC-3' | 5'-AAAGTCGAAGTTCCATCGCTC-3' | 139 |
| **CDKN1B** | 5'-TAATTGGGGCTCCGGCTAACT-3' | 5'-TGCAGGTCGCTTCCTTATTCC-3' | 116 |
| **CDKN1C** | 5'-ACGAGGAGCTGAGTCGTGAG-3' | 5'-CTGTCCACCTCGGTCCACT-3' | 136 |
| **CDKN2A** | 5'-ATGGAGCCTTCGGCTGACT-3' | 5'-GTAACTATTCGGTGCGTTGGG-3' | 108 |
| **CDKN2B** | 5'-GAATGCGCGAGGAGAACAA-3' | 5'-CATCATCATGACCTGGATCGC-3' | 170 |
| **CDKN2C** | 5'-GGGGACCTAGAGCAACTTACT-3' | 5'-CAGCGCAGTCCTTCCAAAT-3' | 81 |
| **CDKN2D** | 5'-AGTCCAGTCCATGACGCAG-3' | 5'-ATCAGGCACGTTGACATCAGC-3' | 87 |
| **CDKN3** | 5'-GGACTCCTGACATAGCCAGC-3' | 5'-CTGTATTGCCCCGGATCCTC-3' | 209 |
| **Angiogenesis pathway-related genes** | | | |
| **ANGPT1** | 5'-TCGTGAGAGTACGACAGACCA-3' | 5'-TCTCCGACTTCATGTTTTCCAC-3' | 173 |
| **ANGPT2** | 5'-ACCCCACTGTTGCTAAAGAAGA-3' | 5'-CCATCCTCACGTCGCTGAATA-3' | 184 |
| **TIE1** | 5'-AAGCAGACAGACGTGATCTGG-3' | 5'-GCACGATGAGCCGAAAGAAG-3' | 184 |
| **TIE2** | 5'-CAGGATACGAACCATGAAGATGC-3' | 5'-GGGGCACTGAATGGATGAAG-3' | 167 |
| **VEGFA** | 5'-AGGGCAGAATCATCACGAAGT-3' | 5'-AGGGTCTCGATTGGATGGCA-3' | 75 |
| **VEGFR1** | 5'-GAAAACGCATAATCTGGGACAGT-3' | 5'-GCGTGGTGTGCTTATTTGGA-3' | 173 |
| **VEGFR2** | 5'-GTGATCGGAAATGACACTGGAG-3' | 5'-CATGTTGGTCACTAACAGAAGCA-3' | 124 |
| **Other genes** | | | |
| **ATP5F1E** | 5'-GGCTGGACTCAGCTACATCC-3' | 5'-TTACGTTGCTGCCAGAAGTCT-3' | 110 |
| **ATP5PF** | 5'-GTTCTCCTCTGTCATTCGGTCA-3' | 5'-TCCAGATGTCTGTCGCTTAGAT-3' | 151 |
| **CALD1** | 5'-TGGAGGTGAATGCCCAGAAC-3' | 5'-GAAGGCGTTTTTGGCGTCTTT-3' | 138 |
| **COL4A1** | 5'-GGGATGCTGTTGAAAGGTGAA-3' | 5'-GGTGGTCCGGTAAATCCTGG-3' | 113 |
| **COL5A2** | 5'-GACTGTGCCGACCCTGTAAC-3' | 5'-CCTGGACGACCACGTATGC-3' | 155 |
| **COX6A1** | 5'-AGTTGGTGTGTCCTCGGTTTC-3' | 5'-GTGAGAGTCTTCCACATGCGA-3' | 117 |
| **COX6C** | 5'-CCAAAACCTCGGATGCGTG-3' | 5'-AAATCTGCGTATGCCTTCTTTCT-3' | 137 |
| **COX7A2** | 5'-CTCGGAGGTAGTTCCGGTTC-3' | 5'-TCTGCCCAATCTGACGAAGAG-3' | 122 |
| **COX7B** | 5'-CTTGGTCAAAAGCGCACTAAATC-3' | 5'-AAAATCAGGTGTACGTTTCTGGT-3' | 91 |
| **COX7C** | 5'-GGTCCGTAGGAGCCACTATGA-3' | 5'-GTGTCTTACTACAAGGAAGGGTG-3' | 136 |
| **CTHRC1** | 5'-CAATGGCATTCCGGGTACAC-3' | 5'-GTACACTCCGCAATTTTCCCAA-3' | 168 |
| **ETFA** | 5'-GTTTTCTGTCCGTGGAACATCC-3' | 5'-ATTTCTGGTCAAGCCACTCTG-3' | 116 |
| **GFP** | 5'-TCTATATCATGGCCGACAAGC-3' | 5'-GGTGTTCTGCTGGTAGTGG-3' | 110 |
| **HTRA1** | 5'-TCCCAACAGTTTGCGCCATAA-3' | 5'-CCGGCACCTCTCGTTTAGAAA-3' | 119 |
| **IL32** | 5'-TGGCGGCTTATTATGAGGAGC-3' | 5'-CTCGGCACCGTAATCCATCTC-3' | 78 |
| **ITGAV** | 5'-ATCTGTGAGGTCGAAACAGGA-3' | 5'-TGGAGCATACTCAACAGTCTTTG-3' | 136 |
| **LGALS1** | 5'-CTGTGCCTGCACTTCAACC-3' | 5'-CATCTGGCAGCTTGACGGT-3' | 187 |

| | | | |
|---|---|---|---|
| **MGP** | 5'-TCCGAGAACGCTCTAAGCCT-3' | 5'-GCAAAGTCTGTAGTCATCACAGG-3' | 65 |
| **NDUFA1** | 5'-GCGTACATCCACAGGTTCACT-3' | 5'-GCGCCTATCTCTTTCCATCAGA-3' | 90 |
| **NDUFA4** | 5'-ATGCTCCGCCAGATCATCG-3' | 5'-TGCCAGACGCAAGAGATACAG-3' | 111 |
| **NDUFB4** | 5'-ATGTCGTTCCCAAAGTATAAGCC-3' | 5'-GAAGCAGGTACTCTCGTTTCAG-3' | 148 |
| **NDUFS6** | 5'-TTCGGTTTGTAGGTCGTCAGA-3' | 5'-CCATCGCACGCTATCACCC-3' | 106 |
| **SUCLA2** | 5'-GCTGCCTAGTAAATGGTGCTG-3' | 5'-GTTGACCAGAATAGCCAGTACC-3' | 173 |
| **TIMM8B** | 5'-TCACTTCATGGAGTTATGTTGGG-3' | 5'-AGACAATTTTCAGTGCGAGAGTC-3' | 78 |
| **TPM1** | 5'-GCCGACGTAGCTTCTCTGAAC-3' | 5'-TTTGGGCTCGACTCTCAATGA-3' | 160 |
| **UQCRB** | 5'-GGTAAGCAGGCCGTTTCAG-3' | 5'-AGGTCCAGTGCCCTCTTAATG-3' | 200 |
| **UQCRH** | 5'-GAGGACGAGCAAAAGATGCTT-3' | 5'-CGAGAGGAATCACGCTCATCA-3' | 170 |
| **UQCRQ** | 5'-ATCCGCACGTCTTCACTAAAG-3' | 5'-TGGATCTCTCGAACTCTTCAGTC-3' | 132 |
| **VIM** | 5'-GACGCCATCAACACCGAGTT-3' | 5'-CTTTGTCGTTGGTTAGCTGGT-3' | 238 |

**Dataset S1 (separate file).** RT-qPCR quantification results of cellular and exosomal mRNAs.
